# Perinatal Outcomes of Asynchronous Influenza Vaccination, Ceará, Brazil, 2013–2018

**DOI:** 10.3201/eid2709.203791

**Published:** 2021-09

**Authors:** José Q. Filho, Francisco S. Junior, Thaisy B.R. Lima, Vânia A.F. Viana, Jaqueline S.V. Burgoa, Alberto M. Soares, Álvaro M. Leite, Simone A. Herron, Hunter L. Newland, Kunaal S. Sarnaik, Gabriel F. Hanson, Jason A. Papin, Sean R. Moore, Aldo A.M. Lima

**Affiliations:** Federal University of Ceará, Fortaleza, Brazil (J.Q. Filho, F.S. Junior, A.M. Soares, Á.M. Leite, A.A.M. Lima);; Ceará State Health Secretariat, Fortaleza (T.B.R. Lima);; Central Public Health Laboratory of Ceará, Fortaleza (V.A.F. Viana, J.S.V. Burgoa);; University of Virginia School of Medicine, Charlottesville, Virginia, USA (S.A. Herron, H.L Newland, K.S. Sarnaik, G.F. Hanson, J.A. Papin, S.R. Moore)

**Keywords:** severe acute respiratory infection, severe flu syndrome, influenza viruses, pregnancy, childbirth, underweight, premature birth, influenza, vaccines, vaccination, Brazil, viruses, respiratory infections

## Abstract

In Ceará, Brazil, seasonal influenza transmission begins before national annual vaccination campaigns commence. To assess the perinatal consequences of this misalignment, we tracked severe acute respiratory infection (SARI), influenza, and influenza immunizations during 2013–2018. Among 3,297 SARI cases, 145 (4.4%) occurred in pregnant women. Statewide vaccination coverage was >80%; however, national vaccination campaigns began during or after peak influenza season. Thirty to forty weeks after peak influenza season, birthweights decreased by 40 g, and rates of prematurity increased from 10.7% to 15.5%. We identified 61 children born to mothers with SARI during pregnancy; they weighed 10% less at birth and were more likely to be premature than 122 newborn controls. Mistiming of influenza vaccination campaigns adversely effects perinatal outcomes in Ceará. Because Ceará is the presumptive starting point for north-to-south seasonal influenza transmission in Brazil, earlier national immunization campaigns would provide greater protection for pregnant women and their fetuses in Ceará and beyond.

Respiratory infections are a leading cause of disease and death worldwide (*1*,*2*), especially among young children and older adults. However, the adverse effects of respiratory infections on pregnant women and fetal development are understudied, particularly in low- and middle-income countries. Respiratory infections in pregnant women can negatively affect birth outcomes, early childhood growth, and neurodevelopment (*3*).

Influenza epidemics are associated with excess rates of pneumonia, related hospitalizations, and death (*4*). Pregnant women and their infants are at heightened risk for severe influenza (*5*,*6*). In 2020, Regan et al. (*7*) conducted a retrospective cohort study of pregnant women from Australia, Canada, Israel, and the United States; results showed hospitalizations for acute respiratory or febrile illnesses were associated with low birthweight but not small-for-gestational-age births. A prospective cohort study of pregnant women in India, Peru, and Thailand showed influenza during pregnancy is associated with late pregnancy loss and reduced mean birthweight (*8*). A meta-analysis (*9*) found that during the 2009 pandemic of influenza A(H1N1)pdm09, the risk for influenza hospitalization was 2-fold higher for women who were pregnant than those who were not. Children born to mothers infected during pregnancy face potential adverse consequences for physical and neurocognitive development. These consequences resemble the growth and developmental challenges described in children born to undernourished mothers in global settings with high rates of pneumonia and diarrhea (*10*–*14*).

In 2018, Almeida et al. (*15*) revealed that 12 of 27 states in Brazil demonstrate annual seasonal influenza activity. States along the coast generally have seasonal influenza patterns, whereas states in the North and Central West regions exhibit no readily identifiable seasonality, probably because landlocked states might have more complex and difficult to detect transmission patterns. In the semiarid state of Ceará, which has a population of ≈8.8 million persons, peak seasonal influenza activity usually begins in mid-May, before the virus spreads southward (*5*). However, influenza circulation begins as early as mid-March. Fortaleza, the state capital, which has a population of 2.7 million, has seasonal influenza peaks 2–3 months earlier than in South and Southeast Brazil (*16**,**17*). Despite these well-described epidemiologic differences, the entire country uses the same vaccination schedule, which is usually concurrent with or after peak influenza activity in the semiarid region (Figure 1). Because vaccine-acquired immunity against influenza usually develops 2 weeks after immunization, we hypothesized that pregnant women and their fetuses in the semiarid region might not be adequately protected against influenza.

**Figure 1:**
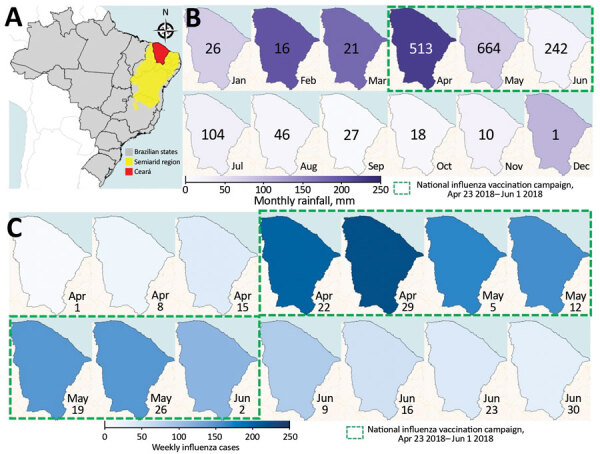
Seasonal patterns in rainfall and influenza and timing of influenza vaccination campaign, Ceará, Brazil, 2018. A) Location of Ceará state in the semiarid region of Brazil. B) Monthly rainfall in Ceará. Numbers indicate total influenza cases each month. C) Weekly influenza cases before, during, and after the annual vaccination campaign in Ceará.

We analyzed whether severe acute respiratory infection (SARI) during pregnancy correlated with low birthweight and premature birth in Ceará. We also evaluated the timing of national influenza vaccine campaigns relative to statewide patterns of SARI and influenza. Finally, we analyzed whether SARI during pregnancy was correlated with low birthweight and prematurity, after adjusting for known confounding variables.

## Methods

### Ethics Approval

We conducted this study with approval from the ethics review committees of the Federal University of Ceará (Fortaleza, Brazil) and the State Health Secretariat (Fortaleza) (registered at Coordenadoria de Gestão do Trabalho e Educação em Saúde (CTGTES)/ Nucleo de Negociação, Valorização e Educação em Saúde NUVEN). We used guaranteed public access information according to the terms of Law No. 12,527 of November 18, 2011. We also used aggregated information from deidentified databases in a manner consistent with the provisions of Conselho Nacional de Saúde Resolution No. 510 of April 7, 2016 (http://www.conselho.saude.gov.br/resolucoes/2016/Reso510.pdf).

### Study Design and Population

Public and private hospitals are required to report SARI cases to the Ministry of Health to inform epidemic prevention, vaccine development, and vaccination campaigns (*18*). We identified SARI cases registered with the Notifiable Diseases Information System (SINAN–Influenza) in Ceará during 2013–2018 (*19*). We defined SARI as onset of fever (even if subjective) accompanied by cough, sore throat, dyspnea, oxygen saturation <95%, or respiratory discomfort within the preceding 7 days (*20*). Previous studies using multivariate regression analysis have shown that cough and fever are the best predictors of laboratory-confirmed influenza (*21*,*22*). We collected data on patient demographics, education, clinical signs and symptoms, epidemiologic risk factors, vaccination status, treatments received, samples collected (i.e., nasopharyngeal secretions, bronchial aspirations, tissue, or others), and reverse transcription PCR (RT-PCR) results from SINAN–Influenza case report forms.

### Molecular Detection of Influenza

RT-PCR detection of influenza viruses was based on a protocol published by the World Health Organization Global Influenza Surveillance Network (*23*). The RT-PCR was specific for the matrix and hemagglutinin genes of seasonal influenza A; B; H1, including A(H1N1)pdm09 and A(H1N1); H3, including A(H3N2); and avian H5 serotypes. Healthcare workers collected patient nasal, oropharyngeal, and nasopharyngeal swab samples and extracted nucleic acid using the QIAamp Viral Mini Kit (QIAGEN, https://www.qiagen.com) according to the manufacturer’s recommended protocols. Laboratory technicians conducted RT-PCR of the extracted viral RNA, enabling production, amplification, and detection of cDNA (*23*).

### Detection of SARI during Pregnancy and Linkage to Birth Data

We constructed a database by linking information from the SINAN–Influenza database with data from the Sistema de Informações Sobre Nascidos Vivos (SINASC) database (*24*). We used MySQL version 5.0.11 (Oracle Corporation, https://www.mysql.com), R version 3.6.2 with the genderBR package 1.1.0 (The R Project for Statistical Computing, https://www.r-project.org), and Stata version 11 (StataCorp LLC, https://www.stata.com) to construct and manage the combined database. We compared SARI case report forms and birth records of pregnant women with documented SARI. Separately, we linked deidentified data from individual pregnant women to birth certificate data for a case–control study. When possible, we also linked influenza test results to these records. We collected each child’s birthweight and Apgar score, as well as information concerning demographics, maternal education, previous and current pregnancies, and mode of delivery from birth certificate data.

### Maternal and Fetal Effects of SARI

To evaluate the effects of maternal SARI on birthweight and gestational length, we designed an observational descriptive study of children born to mothers who did and did not have SARI during pregnancy. The control group was composed of randomly selected children born to mothers matched by age (<3 months) to mothers who had SARI during pregnancy. We collected birthweights from SINAN data recorded during routine clinical practice.

### Annual Periodicity in Birthweight and Gestational Length

To evaluate the effects of seasonal influenza on birth outcomes, we investigated the periodicity associated with birthweight and gestation length in Ceará. We obtained birth outcomes from the SINASC database. SINASC classifies gestational length using a scale of 1–6 in which 1 indicates <22 weeks, 2 indicates 22–27 weeks, 3 indicates 28–31 weeks, 4 indicates 32–36 weeks, 5 indicates 37–41 weeks, and 6 indicates *>*42 weeks of gestation. We defined preterm birth as <37 weeks’ gestation. We calculated the average birthweights and gestations by epidemiologic week.

### Sample Size and Statistical Analysis

**Figure 2:**
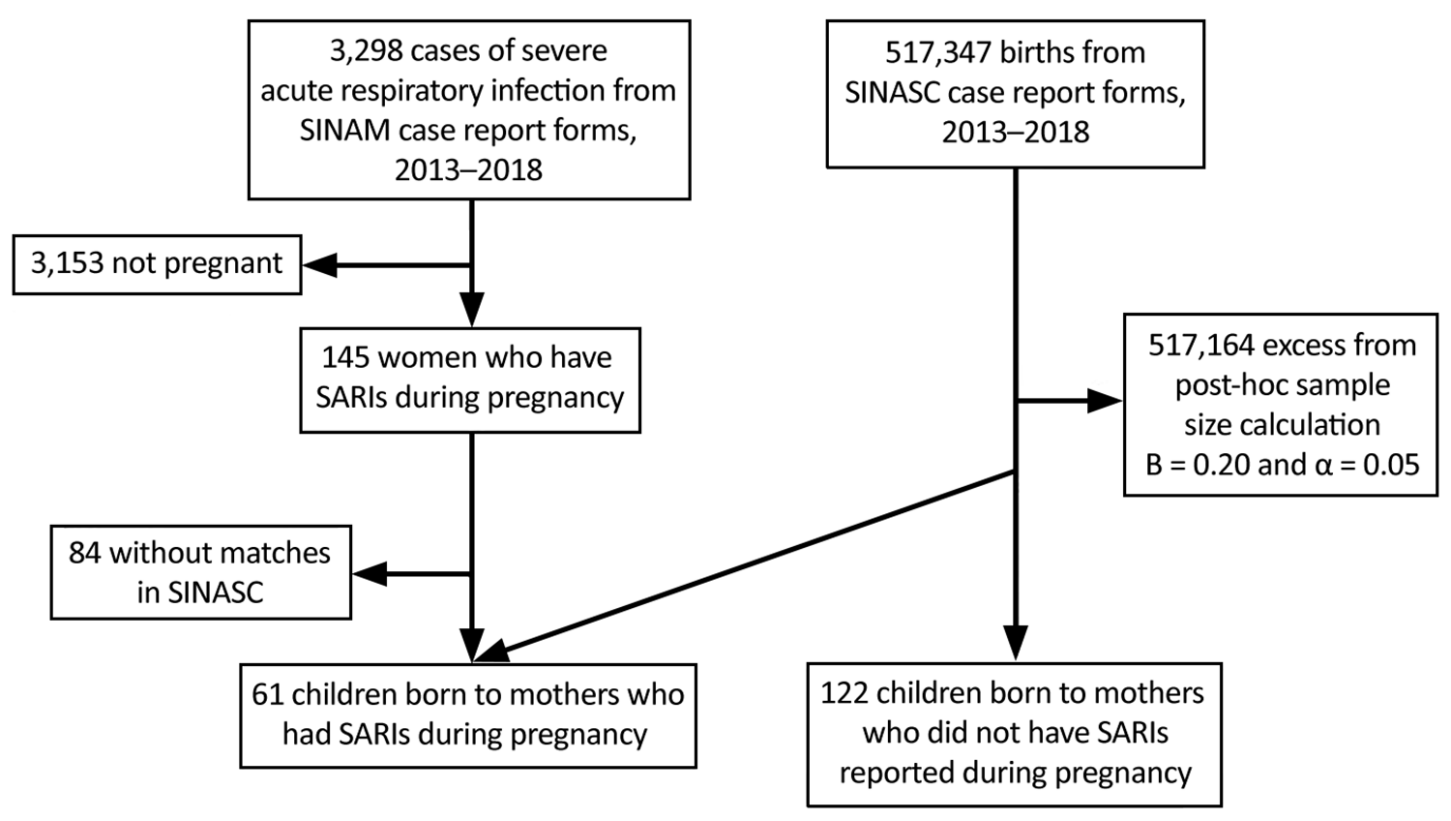
Design of study of SARI during pregnancy, Ceará, Brazil, 2013–2018. SARI, severe acute respiratory infection; SINASC, the Sistema de Informações Sobre Nascidos Vivos (*24*); SINAN, Notifiable Diseases Information System (*19*).

We estimated the sample size needed to detect an effect of SARI on birthweight would be 183 children: 61 born to mothers who did and 122 born to mothers who did not have SARI during pregnancy (Figure 2). This sample size provided a statistical power of 80% at p<0.05 for children who were 10% underweight compared with controls (*21*,*23*). We compared mean birthweight using the formula n_1_ = (*u* + *v*)^2^(σ_1_^2^ + σ_2_^2^/*K*)/(µ_1_ – µ_2_)^2^, where µ_1_ – µ_2_ represents the difference between means, σ_1_ and σ_2_ represent SDs, *u* represents the 1-sided percentage point of the normal distribution corresponding to 100% (e.g., if power = 80%, then *u* = 0.84), *v* represents the percentage point of the normal distribution corresponding to the 2-sided significance level (e.g., if significance level = 5%, then *v* = 1.96), and *K* = n_2_/n_1_. We used ClinCalc.com (https://clincalc.com/Stats/SampleSize.aspx) for sample size calculations.

Data were entered into spreadsheets and checked by 2 independent researchers to ensure accuracy. All data were deidentified. We conducted statistical analysis using SPSS Statistics 20.0 (IBM Corporation, https://www.ibm.com). We used the Shapiro-Wilk test to evaluate normality of quantitative data and the Levene test to evaluate equality of variances. For nonparametric variables, we used the Mann-Whitney test. We analyzed qualitative variables using the χ^2^ test or Fisher exact test. We used GraphPad Prism version 3.0 for Windows (GraphPad Software, https://www.graphpad.com) for complementary statistical analysis, table formatting, and figure creation. We used adjusted and nonadjusted multivariate logistic regression models to assess underweight and preterm birth associations. To reduce possible influence from confounding variables, we coadjusted variables measuring sex, maternal education, information from previous and current pregnancies, and delivery data. We used odds ratios or relative risk ratios with 95% CIs to assess the relationship between a variable and its outcome. All statistical tests were 2-sided with a significance level of p<0.05.

## Results

Using the SINAN database, we identified 3,298 SARI cases in Ceará during 2013–2018, including 145 cases among pregnant women (Table 1). We linked the SINAN and SINASC databases to identify 61 children born to mothers who had >1 SARI during pregnancy. We used the same databases to identify 122 children born to age-matched pregnant women who did not have recorded SARI during pregnancy. 

**Table 1:** Prevalence of severe acute respiratory infection, Ceará, Brazil, 2013–2018*

Variable	Year					
2013	2014	2015	2016	2017	2018
Sex†						
M	141 (43)	68 (39)	115 (40)	272 (50)	150 (53)	854 (51)
F	189 (57)	105 (61)	174 (60)	274 (50)	135 (47)	820 (49)
Age, y (range)‡	26.02 (0–96)	26.54 (0–97)	23.11 (0–94)	16.85 (0–100)	1.58 (0–94)	5.26 (0–102)
Age groups at high risk†						
<6 mo	47 (14)	46 (27)	76 (26)	52 (10)	88 (31)	235 (14)
6 mo to 5 y	43 (13)	16 (9)	23 (8)	174 (32)	84 (29)	594 (35)
>60 y	53 (16)	27 (16)	30 (10)	102 (19)	21 (7)	247 (15)
Pregnant women†	38 (12)	13 (8)	32 (11)	17 (3)	10 (4)	35 (2)
SARI cases, total†	330 (100)	173 (100)	289 (100)	546 (100)	285 (100)	1,674 (100)
Influenza	56 (17)	24 (14)	58 (20)	107 (20)	36 (13)	451 (27)
Noninfluenza	61 (18)	22 (13)	36 (12)	64 (12)	101 (35)	21 (1)
Unspecified or unknown	213 (65)	127 (73)	198 (69)	375 (69)	148 (52)	1,202 (72)
Influenza subtypes§	56 (100)	24 (100)	58 (100)	107 (100)	35 (100)	450 (100)
Seasonal A(H1N1)	30 (54)	18 (75)	1 (2)	89 (83)	2 (6)	309 (69)
Other seasonal A(H1)	0	0	45 (78)	0	1 (3)	0
Seasonal A(H3)	2 (4)	0	0	0	25 (71)	23 (5)
A, unknown subtype	22 (39)	1 (4)	4 (7)	16 (15)	0	14 (3)
B	2 (4)	5 (21)	8 (14)	2 (2)	7 (20)	104 (23)
SARI deaths¶	13 (100)	2 (100)	1 (100)	40 (100)	24 (100)	159 (100)
Influenza	9 (69)	1 (50)	0	17 (43)	5 (21)	75 (47)
Death rate of laboratory-certified influenza#	16.1	4.2	0	15.9	20.8	16.6
Other viruses/etiologic agents or unspecified	4 (31)	1 (50)	1(100)	23 (58)	19 (79)	84 (53)
Influenza vaccination coverage**	88	84	83	91	90	NA

*Data from Notifiable Diseases Information System (*19*). Values are no. (%), except as indicated. NA, not available; SARI, severe acute respiratory infection. †Of total SARI patients. ‡Values are median age (range). §Of total persons with identified influenza subtype. ¶Of total SARI deaths. #Of total laboratory-certified influenza deaths. **Among persons at risk (estimated at ≈2.6 million). Population at risk comprises children <6 mo of age; children 6 mo to <5 y of age; persons >60 years of age; pregnant women; postpartum women (<45 d after delivery); healthcare workers; teachers; indigenous persons; persons who have chronic noncommunicable diseases and other immunocompromising conditions; persons 12–21 y of age experiencing poverty; prisoners; prison system officials; and military police, civilians, firefighters, and armed forces.

**Figure 3:**
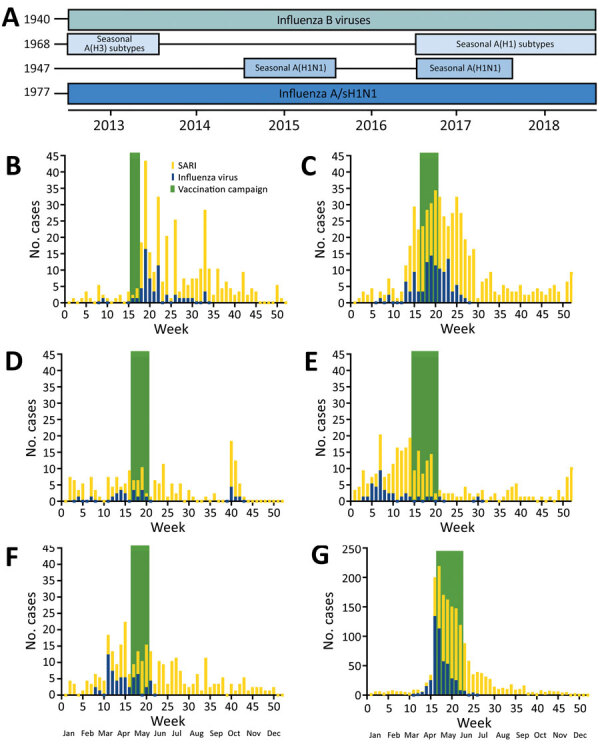
Patterns of influenza and severe acute respiratory infections and timing of influenza vaccination campaigns, Ceará, Brazil, 2013–2018. A) Dominance of various influenza subtypes over time. Years indicate date each strain was first identified. B) Weekly cases of influenza and severe acute respiratory infections.

We observed equal proportions of SARI cases among male and female patients registered in the SINAN database of 3,298 overall SARI cases in Ceará. Children <5 years of age comprised 27%–61% of patients; children <6 months of age comprised 10%–31% of patients. Older adults (7%–32%) and pregnant women (2%–38%) also comprised large proportions of patients. We observed cases of seasonal H1N1 throughout the study period, notably in 2013 (54%), 2014 (75%), 2016 (83%), and 2018 (69%). The highest number of SARI cases occurred in 2018, mostly caused by seasonal H1N1 and influenza B viruses (23%). We observed sporadic cases of seasonal influenza caused by other H1 subtypes in 2015 and 2017 and seasonal H3 subtypes in 2013 and 2017–2018 (Table 1; Figure 3, panels A, B). Influenza death rates varied from 0%–21%; the peak death rate occurred during a season predominated by H3 subtypes.

The median age of pregnant women who had SARI was 26 years (range 15–44 years). Among 145 pregnant women who had SARI, 43 (32%) had laboratory-confirmed influenza. Among those 43 women, 42% had illnesses caused by H1N1, 33% by H3 subtypes, 7% by influenza A viruses without an identified subtype, and 19% by influenza B subtypes (19%). We identified no deaths caused by SARI in pregnant women (Table 2).

**Table 2:** Characteristics of pregnant women with SARI, Ceará, Brazil, 2013–2018*

Characteristic	Value
Total SARI cases	3,297 (100)
Among pregnant women	145 (4)
Median age, y (range)	25.95 (15–44)
SARI cases with etiologic testing	134 (100)
Influenza	43 (32)
Noninfluenza	11 (8)
Unspecified or unknown	80 (60)
% Laboratory-confirmed influenza	32.1
Influenza subtypes†	43 (100)
Seasonal A(H1N1)	18 (42)
Other seasonal A(H1)	0
Seasonal A(H3)	14 (33)
A (unknown subtype)	3 (7)
B	8 (19)
SARI deaths‡	3 (100)
Influenza	0
Death rate of laboratory-certified influenza	0
Other viruses/etiologic agents or unspecified	3 (100)

*Data from Notifiable Diseases Information System (*19*). Values are no. (%), except as indicated. SARI, severe acute respiratory infection. †Of total persons with identified influenza subtype. ‡Of total SARI deaths.

**Figure 4:**
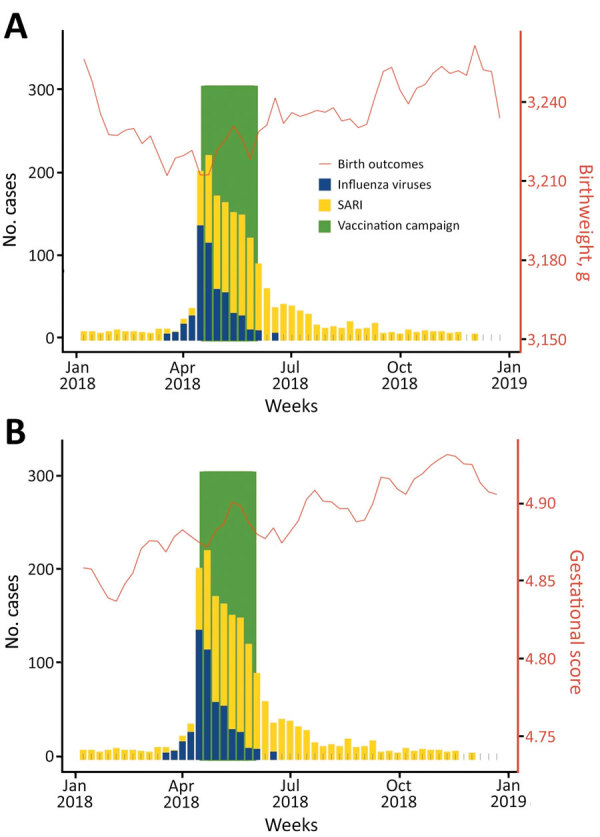
Associations between SARI among pregnant women and birth outcomes, Ceará, Brazil, 2018. A) By birthweight; B) by gestational score. Gestational length scored using a 1–6 scale in which 1 indicates <22 weeks, 2 indicates 22–27 weeks, 3 indicates 28–31 weeks, 4 indicates 32–36 weeks, 5 indicates 37–41 weeks, and 6 indicates >42 weeks of gestation. SARI, severe acute respiratory infection.

To better visualize the relationship between birth outcomes, SARI, and influenza, we overlaid sets of data for 2018 on the same plot (Figure 4, panel A). We found that average birthweight decreased shortly before influenza season. During 2018, birthweight peaked in the first week of the year. By week 15, average birthweight had fallen by ≈40 g (Figure 4, panel A). After the influenza vaccination campaign ended, SARI cases declined and birthweights returned to their yearly averages. For all years of the study, we found lower average gestational scores, which indicates a higher proportion of preterm births, before and during influenza season (Figure 4, panel B).

**Figure 5:**
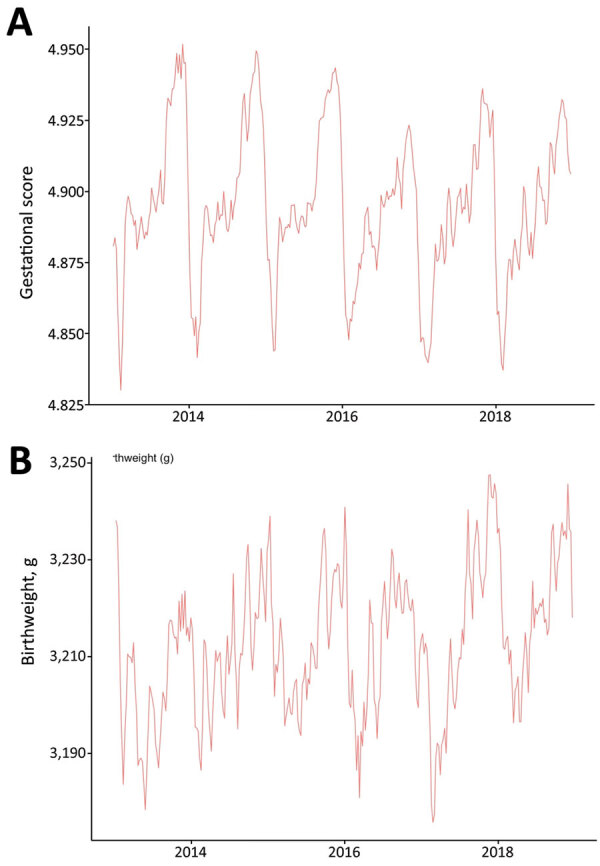
Seasonal periodicity of gestational score (A) and birthweight (B), Ceará, Brazil, 2013–2018. Gestational length scored using a scale of 1–6: 1 indicates <22 weeks, 2 indicates 22–27 weeks, 3 indicates 28–31 weeks, 4 indicates 32–36 weeks, 5 indicates 37–41 weeks, and 6 indicates >42 weeks of gestation.

Each year, average birthweights oscillated by up to 40 g, or 1%–2% of total birthweight (Figure 5). In February, a month associated with worse birth outcomes, 15.5% (8,399/54,311) of children were born prematurely (<37 weeks), whereas in October, a month associated with better birth outcomes, 10.7% (6,552/61,067) of children were born prematurely. These data indicate that circannual oscillations in birth outcomes might be associated with SARI and seasonal influenza in Ceará.

Children born to mothers who had SARI during pregnancy had significantly lower birthweights (p = 0.02), higher risk for prematurity (p = 0.03), shorter gestation times (p<0.01), and lower Apgar scores at 5 minutes after birth than children in the control group (p<0.01). Mothers who had SARI during pregnancy had significantly less formal education than mothers who did not have SARI (p<0.01). Mothers with SARI had a significantly lower number of previous pregnancies (p = 0.01), previous vaginal births (p<0.01), and previous live births (p = 0.01). Mothers with SARI had a higher number of previous cesarean sections (p<0.01). Cesarean deliveries and medical assistance were more frequent in cases versus controls (86.7% vs. 0.8%; p<0.01) (Table 3).

**Table 3:** Characteristics of children born to women who did and did not have severe acute respiratory infections during pregnancy, Ceará, Brazil, 2013–2018*

Variables	Total	Born to mothers who had severe acute respiratory infections during pregnancy		p value†
		Yes	No	
Total	183	61	122	
Sex				
M	81 (44)	24 (39)	57 (47)	0.20
F	102 (56)	378 (61)	65 (53)	
Mean birthweight, g (SD)	3,090.1 (665.42)	2,879.1 (783.57)	3,195.6 (572.61)	0.02
Preterm birth (i.e., gestation <37 wks)	26 (14)	16 (27)	10 (13)	0.03
Mean Apgar index (SD)				
At 1 min	8.0 (1.45)	7.9 (1.66)	8.0 (1.32)	0.83
At 5 min	9.0 (0.98)	8.9 (0.75)	9.1 (1.08)	<0.01
Mean maternal age, y (SD)	28.3 (6.65)	28.3 (6.69)	28.3 (6.66)	0.98
Education				<0.01
None	0	0	0	
Elementary I: <4th grade	8 (4.6)	1 (1.7)	7 (6.0)	
Elementary II: <8th grade	19 (10.9)	1 (1.7)	18 (15.5)	
Secondary: <12th grade	60 (34.5)	14 (24.1)	46 (39.7)	
Partial college	73 (42.0)	34 (58.6)	39 (33.6)	
College	14 (8.0)	8 (13.8)	6 (5.2)	
Previous pregnancies				
Median no. previous pregnancies (range)	2 (0–16)	1 (0–7)	2 (0–16)	0.01
Median no. vaginal deliveries (range)	2 (0–12)	0 (0–4)	2 (0–12)	<0.01
Median no. cesarean sections (range)	0 (0–4)	0 (0–4)	0 (0–2)	<0.01
Median no. live births (range)	2 (0–12)	1 (0–7)	2 (0–12)	0.01
Median no. fetal losses or abortions (range)	0 (0–4)	0 (0–2)	0 (0–4)	0.16
Current pregnancy				
Mean gestation length, wks (SD)	37.8 (3.15)	36.9 (3.72)	38.4 (2.50)	<0.01
Mean no. prenatal consultations (SD)	6.9 (3.15)	7.0 (2.55)	6.7 (2.16)	0.56
Mean start of prenatal care started, mo (SD)	2.9 (1.38)	2.8 (1.22)	3.1 (1.48)	0.14
Type of pregnancy				0.11
Single	181 (98.9)	59 (96.7)	122 (100)	
Twins	2 (1.1)	2 (3.3)	0	
Triplets or more	0	0	0	
Fetal presentation at delivery‡				0.09
Cephalic	138 (96.5)	54 (93.1)	84 (98.8)	
Pelvic or podalic	5 (3.5)	4 (6.9)	1 (1.2)	
Transversal	0	0	0	
Induced labor				0.10
Y	5 (3.6)	4 (6.9)	1 (1.2)	
N	134 (96.4)	54 (93.1)	80 (98.8)	
Type of delivery§				<0.01
Vaginal	128 (70.7)	8 (13.3)	120 (99.2)	
Cesarean	53 (29.3)	52 (86.7)	1 (0.8)	
Cesarean section without labor¶				NS
Y	29 (76.3)	29 (76.3)	0	
N	9 (23.7)	9 (23.7)	0	
Birth attendant#				0.01
Doctor	122 (84.1)	59 (98.3)	63 (74.1)	
Obstetric nurse	6 (4.1)	1 (1.7)	5 (5.9)	
Midwife	9 (6.2)	0	9 (10.6)	
Others	8 (5.5)	0	8 (9.4)	

*Data from Sistema de Informações Sobre Nascidos Vivos (*24*). Values are no. (%), except as indicated. NS, not significant. †Mann-Whitney test was used for variables whose distribution was not normal and χ2 analysis was used for normally distributed data. ‡Of 143 cases with available data. §Of 181 cases with available data. ¶Of 38 cases with available data. #Of 135 cases with available data.

**Figure 6:**
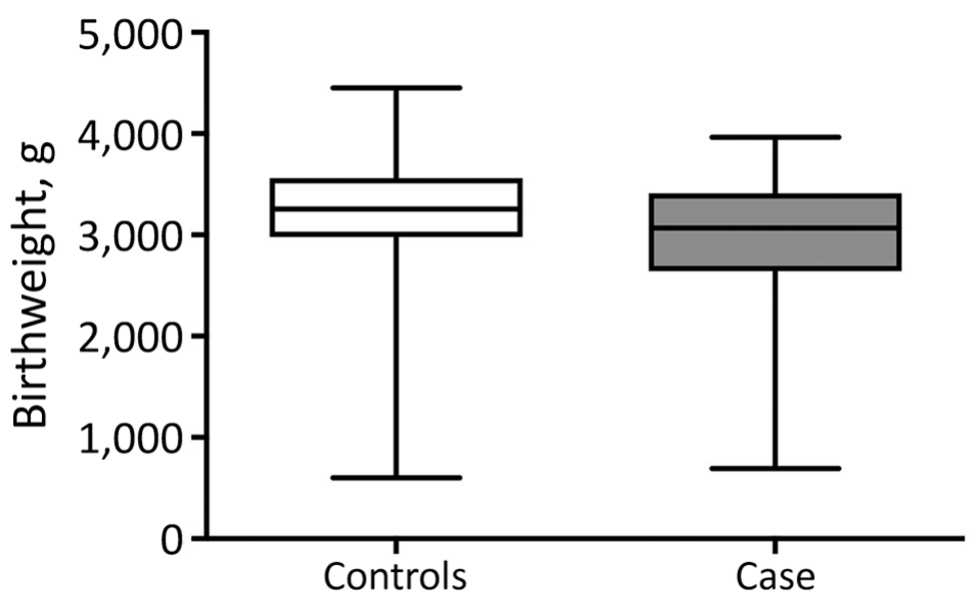
Comparison of birthweights of children born to mothers who did (cases) and did not (controls) have severe acute respiratory infections during pregnancy, Ceará, Brazil, 2013–2018. Horizontal lines within boxes indicate means; box tops and bottoms indicate 25th and 75th percentiles; whiskers indicate 95% CIs. p = 0.02 by Mann-Whitney test.

We used multiple logistic regression to identify predictor variables independently associated with SARI during pregnancy. First, we examined 11 significant variables identified by univariate analysis (Table 3), of which 5 showed >40% collinearity. We had an adequate sample size (123 cases) to run a logistic regression for these 5 variables (*23*,*25*). The overall model fit showed a χ^2^ value of 23.135 (df = 6; p<0.01). The Cox and Snell test and Nagelkerke test indicated variances between 17.1% and 23.2%. Including predictor variables increased model accuracy from 61% to 68%. We found that birthweight (p = 0.03) and attendance of birth by a physician (p = 0.04) were significantly associated with SARI during pregnancy (Table 4; Figure 6).

**Table 4:** Odds ratios for characteristics of 61 children born to women who had SARI during pregnancy compared with 122 children born to women who did not have SARI, Ceará, Brazil, 2013–2018*

Variables	Odds ratio (95% CI)	Adjusted odds ratio (95% CI)*
Birthweight, g†	0.999 (0.999–1.000)	0.999 (0.998–1.000)
Preterm birth (i.e., gestation <37 wks)‡	2.944 (1.100–7.879)	0.849 (0.151–4.771)
Mother education	4.320 (1.095–17.051)	1.156 (0.198–6.746)
No. previous pregnancies¶	0.795 (0.659–0.960)	0.894 (0.727–1.099)
No. wks gestation†	0.852 (0.756–0.961)	1.025 (0.794–1.325)
Birth attended	20.603 (2.692–157.697)	9.327 (1.144–76.060)

*Data from Sistema de Informações Sobre Nascidos Vivos (*24*). SARI, severe acute respiratory infection. *Determined by multivariate logistic regression analysis of variables associated with births requiring the presence of a skilled attendant. †Analysis of means and SDs. ‡Categories comprise children born at <37 and >37 wks gestation. §Categories comprise mothers who had attended no schooling, elementary I (1st–4th grade), elementary II (5th–8th grade), secondary school (9th–12th grade), incomplete college, and complete college. Analysis grouped no schooling, elementary I (1st–4th grade), elementary II (5th–8th grade), and secondary school (9th–12th grade), as well as incomplete and complete higher education categories. ¶Analysis of medians and variations. #Categories comprise births attended by a physician, obstetric nurse, midwife, or other. Analysis grouped obstetric nurse, midwife, and other categories.

## Discussion

We documented 3,298 SARI cases in Ceará, Brazil, during 2013–2018. Cases occurred predominantly in younger children, especially children <6 months of age, as well as older adults and pregnant women. These data are consistent with previous studies showing higher rates of infection among younger populations but increased death rates among older adults (*26*).

H1N1 was the dominant influenza subtype during seasonal epidemic outbreaks, illustrating the capacity of this strain to recirculate and co-circulate with other seasonal influenza strains (Table 1; Figure 3, panel A). H1N1 caused a high death rate throughout the study. However, in 2015, when seasonal H1 strains predominated in Ceará, we observed a lower overall death rate among influenza patients. These data are consistent with prior literature showing more deaths associated with H1N1 (*27*). The mortality rate in our study might be attributable to the mistiming of vaccination campaigns, which occurred during and after peak influenza activity in Ceará. The state has high vaccination coverage, suggesting that earlier administration of influenza vaccines might reduce death and disease. Mistiming of immunization schedules also might explain the unusually high disease incidence among infants <6 months of age. The World Health Organization does not recommend immunization in this age group. Consequently, immunization of pregnant mothers is especially crucial for passive immunity against influenza during the first 6 months of life.

In this study, we assessed whether SARI during pregnancy was associated with a higher risk for low birthweight or prematurity. We found statewide correlations between peak influenza activity and nadirs in birthweight and gestational length. Furthermore, we confirmed associations of maternal SARI with low birthweight and preterm birth in matched mother–infant pairs. These associations remained significant when adjusted for confounders by multiple logistic regression. Our findings agree with 2 recently published studies (*7*,*8*) showing an association of SARI, including influenza, among pregnant women with low birthweights. Pregnant mothers who had SARI were more likely to require medical assistance during labor than those who did not have SARI.

Our study aligns with earlier reports showing the importance of prevention and adjustment of influenza vaccine campaign schedules to avoid complications of influenza (*6*,*28*–*31*). Previous studies show the importance of the first 1,000 days of life in reducing undernutrition, enteric infections, and risk for metabolic syndrome and cardiovascular diseases (*32*,*33*). Neurocognitive, physical, and educational deficits have been well-documented among children exposed in utero or during the first months of life to influenza and other diseases such as enteric infections (*10*–*12*,*34*).

The first limitation of our study is that we analyzed only cases of influenza associated with SARI and did not include cases of mild-to-moderate influenza. However, our analyses of statewide birth outcomes detected substantial periodicity in birthweights and gestational length; poorer outcomes coincided with influenza season. Maternal influenza also might affect other perinatal outcomes, such as medical necessity for caesarean birth. Second, our nested observational descriptive study cannot infer a causal relationship between maternal SARI and adverse birth outcomes. However, the associations were robust to logistic regression adjusted for several potential confounders. In addition, because hospitalization is part of the case definition for SARI, public and private hospitals (but not private clinics) are required to report SARI cases to SINAN. Although many private clinics do report, most reported cases come from public institutions. Thus, we might not have analyzed all SARI cases in Ceará. Finally, our results suggest that asynchronous vaccination schedules might be associated with adverse influenza outcomes in Ceará, but we did not model the extent to which earlier immunization or the use of vaccine strains from the Northern or Southern Hemispheres might mitigate these outcomes. Recent epidemiologic models suggest Ceará is the starting point for influenza transmission from the semiarid region in southern Brazil, hence earlier immunization in Ceará might have substantial benefits for the region and country (*5*). We did not account for infections with Zika virus as a potential confounder of our findings because the reported Zika incidence was 0 during 2013–2016; however, testing for Zika was not routinely performed during this time period. The state had low Zika incidence during the study: 5.6 cases/100,000 persons in 2017 and 0.2 cases/100,000 persons in 2018 (*35*).

In conclusion, our results show that late timing of influenza vaccination in Ceará, a populous semiarid state in Brazil with high vaccination coverage, correlates with adverse perinatal outcomes. In addition, we found that mean birthweight and rates of prematurity followed an annual periodicity, suggesting additional associations with seasonal influenza. Finally, we confirmed a robust association of maternal SARI with poor birth outcomes using an observational descriptive study design. Further work is urgently needed to model and study the optimal timing, potential impact, logistics, economics, and implementation of such a diversified national influenza vaccine strategy. Because Ceará is the presumptive starting point for an annual north-to-south pattern of seasonal influenza transmission in Brazil (*15*), our data indicate earlier timing of national immunization campaigns, ideally before seasonal influenza circulation in Ceará, might provide substantial benefits not only for women and children in the semiarid region but also for Brazil as a country. 
